# Dietary fiber‐based oral nanovaccine promotes gut‐to‐nose trafficking of regulatory cells and drives antigen‐specific tolerance to relieve airway allergy

**DOI:** 10.1002/imt2.70158

**Published:** 2026-07-25

**Authors:** Yuting Qin, Zeming Wang, Yuru Zong, Zefang Lu, Junxiu Liu, Ruifang Zhao, Guangjun Nie, Hanqing Chen

**Affiliations:** ^1^ Beijing Key Laboratory for Drug Delivery Nanocarriers, CAS Center for Excellence in Nanoscience National Center for Nanoscience and Technology Beijing China; ^2^ Institute of Nanotechnology And Intelligence (inAI) Jinan University Guangzhou China; ^3^ Center of Materials Science and Optoelectronics Engineering University of Chinese Academy of Sciences Beijing China; ^4^ Beijing Key Laboratory of Environment and Aging, Department of Nutrition and Food Hygiene, School of Public Health Capital Medical University Beijing China; ^5^ Department of Otolaryngology‐Head and Neck Surgery Peking University First Hospital Beijing China

## Abstract

Current therapies for allergic rhinitis primarily provide symptomatic relief but fail to establish durable antigen‐specific immune tolerance. Here, we developed an oral nano‐dietary fiber (NDF) platform that integrates allergen delivery with microbiota‐driven immunomodulation, using dextran as both an antigen carrier and a fermentable substrate for intestinal bacteria. NDF enabled coordinated allergen release and sustained short‐chain fatty acid (SCFA) production in the gut, promoting SCFA–FFAR2‐dependent expansion of regulatory B and T cells and their trafficking to the nasal mucosa. In murine models, NDF slowed disease progression, improved airway function, and reduced allergen‐specific IgE levels. Together, these findings establish NDF as an oral tolerogenic vaccine that provides durable protection against allergic airway disease through the gut‐nasal axis.

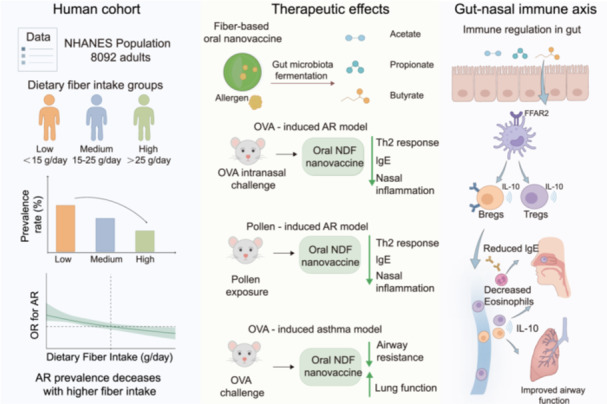

To the Editor,

Allergic rhinitis (AR) affects over 400 million people worldwide, and its prevalence continues to rise with urbanization, dietary transition, and microbiome perturbations [1, 2]. Current therapies, including antihistamines, intranasal corticosteroids, or sublingual immunotherapy (SLIT), can alleviate symptoms, but often fail to induce durable antigen‐specific immune tolerance [3, 4]. SLIT remains the only approved allergen‐specific immunotherapy for AR, yet its clinical benefit is hindered by poor patient compliance, prolonged treatment courses, and local adverse reactions [4, 5]. There is an urgent need to develop an innovative pathophysiology‐oriented and patient‐friendly strategy to restore long‐term mucosal tolerance in patients with AR.

AR is characterized by the breakdown of mucosal tolerance and the development of allergen‐specific type 2 helper T‐cell (Th2)‐driven inflammation [6, 7, 8]. Emerging evidence highlights the gut–nose axis, where gut microbiota ferment dietary fibers into short‐chain fatty acids (SCFAs) that expand regulatory T cells (Tregs) and regulatory B cells (Bregs) through receptors such as free fatty acid receptor 2 (FFAR2) [7, 8, 9, 10]. Epidemiological evidence also supports a protective association between dietary fiber intake and allergic diseases [11]. However, direct SCFA supplementation is limited by inconsistent bioavailability, rapid absorption, and off‐target effects. Recently, targeting the gut microenvironment has evolved into a highly precise therapeutic modality [12, 13, 14, 15]. Building on this rationale, we developed an oral and fermentable nano‐dietary fiber (NDF) platform that functions as a therapeutic tolerance‐inducing vaccine [16, 17, 18], demonstrating that it drives SCFA‐dependent trafficking of IL‐10‐producing Tregs and Bregs from the gut to the nasal mucosa, effectively relieving pollen‐induced AR, asthma comorbidity, and long‐term immune memory.

## HIGHER DIETARY FIBER INTAKE CORRELATES WITH LOWER AR PREVALENCE IN HUMANS

To explore the relationship between dietary fiber intake and AR prevalence, we first analyzed data from 8092 adults in the NHANES cohort (Figure S1A). AR prevalence decreased progressively with higher fiber intake (Figure S1B). Compared to the high‐intake group (>25 g/day), the low‐intake group (<15 g/day) showed a significantly higher association with AR (odds ratio [OR] = 1.39, 95% CI: 1.16–1.66, *p* < 0.001) (Figure S1C and Tables S1, 2). Multivariable logistic regression adjusted for age and sex confirmed that lower fiber intake was associated with increased AR prevalence (Figure S1D and Table S3). Moreover, each 10 g/day increase in dietary fiber intake was associated with a 13.4% reduction in AR prevalence (Figure S1E and Table S4), and restricted cubic spline analysis demonstrated a linear inverse association (Figure S1F). Collectively, these population‐based data support an association between insufficient dietary fiber intake and increased AR prevalence.

## NDF ENABLES SUSTAINED ALLERGEN DELIVERY AND IN SITU SCFA PRODUCTION

**FIGURE 1:**
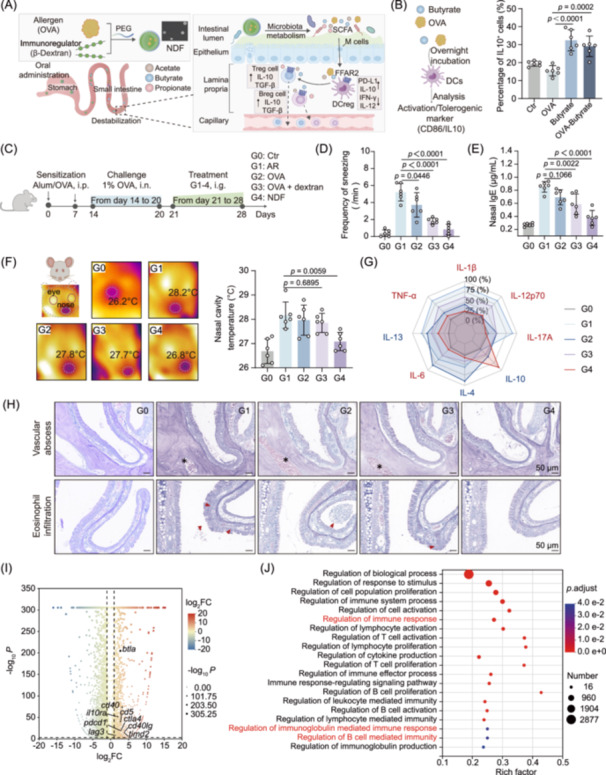
Oral administration of nano‐dietary fiber (NDF) induces immune tolerance and alleviates allergic rhinitis through modulation of the intestinal immune microenvironment. (A) Schematic illustrating the synthesis of NDF and the proposed mechanism of oral tolerance induction. OVA was added to a mixture of dextran and PEG and encapsulated in NDF via freezing‐induced aqueous phase separation. Orally administered NDF are metabolized by intestinal microbiota into SCFAs, which exert immunoregulatory effects. The released OVA protein, as an allergen, is proposed to induce oral tolerance in this SCFA‐rich microenvironment. (B) DCs were incubated overnight with OVA, butyrate, or OVA‐butyrate mixture and subsequently analyzed by flow cytometry for activation and tolerogenic markers. Quantification of IL‐10‐producing DCs is shown on the right. Treatment with butyrate or OVA‐butyrate significantly increased the frequency of IL‐10^+^ DCs compared with OVA alone, indicating enhanced acquisition of a tolerogenic phenotype. (C) Schematic of the experimental timeline for allergic rhinitis induction and treatment. BALB/c mice were sensitized by intraperitoneal injection of aluminum hydroxide (alum) and OVA using an 8‐day escalating‐dose oral regimen (0.5 to 4 mg OVA per mouse) on Days 0 and 7. From Day 14 to Day 20, mice received daily intranasal challenges with 1% OVA (10 µL per nostril). Therapeutic oral gavage with PBS, free OVA, free OVA mixed with dextran (OVA + dextran), or NDF was administered from Day 21 to Day 28. During this treatment phase, the OVA dose was escalated from 0.5 mg to 4 mg per mouse, while the dextran dose was maintained at 4 mg per mouse. I.p., intraperitoneal administration; i.n., intranasal administration; i.g., intragastric administration. (D) Sneezing frequency recorded for 15 min after the third intranasal challenge (Day 31). (E) Levels of OVA‐specific IgE in nasal wash fluid after the final challenge. (F) Representative infrared thermographic images of the nasal region (left) and quantification of nasal surface temperature (right) following allergen challenge. (G) Radar plot of representative cytokines in nasal lavage fluid across experimental groups. Th1‐associated cytokines (IL‐12p70, TNF‐α, IL‐1β, IL‐6 and IL‐17A) and Th2‐associated cytokines (IL‐10, IL‐4 and IL‐13) were included to visualize group‐specific immune profiles. Cytokine levels were normalized by z‐score transformation. G1 and G2 exhibited a predominantly pro‐inflammatory cytokine profile, whereas G4 showed a tolerogenic profile, with increased IL‐10 expression relative to the other groups. (H) Representative Giemsa‐stained sections of nasal mucosa tissue. Asterisks indicate vascular abscess, and red arrows highlight eosinophil infiltration. Scale bar, 50 µm. (I) Volcano plot of RNA‐seq data comparing the intestinal transcriptome of AR mice and NDF‐treated mice. Select immunoregulatory genes (e.g., *cd40*, *ctla4*, *pdcd1*, *timd2*) are highlighted. (J) GO term enrichment analysis of genes upregulated by NDF treatment. The bubble plot shows significantly enriched biological processes related to immune regulation. Data in (B), (D), (E), and (F) are presented as mean ± SD (*n* = 6). Data were analyzed using one‐way ANOVA with post hoc Tukey's test.

To translate this protective association into a therapeutic strategy, we developed a NDF platform based on high‐molecular‐weight β‐dextran to encapsulate the model allergen OVA via a freezing‐induced aqueous phase separation method (Figure 1A). β‐dextran is stable under gastric conditions, fermentable by intestinal microbiota, and suitable for oral antigen delivery [19]. As shown in SEM images and DLS data, the optimized NDF formulation (dextran‐to‐OVA ratio = 1:1) displayed uniform spherical morphology, an average hydrodynamic diameter of 89 ± 8.9 nm, a low polydispersity index of 0.021, a zeta potential of −5.1 ± 1.3 mV, and an encapsulation efficiency exceeding 96% (Figure S2A,B). In vivo fluorescence imaging showed that NDF prolonged intestinal retention for more than 16 h compared with free OVA (Figure S2C). After oral administration, fecal SCFAs, including acetate, propionate, butyrate, and isobutyrate, were significantly increased (Figure S2D), indicating efficient microbial fermentation of NDF. Transcriptomic analysis of intestinal tissues revealed enrichment of pathways associated with differentiation of regulatory T cells and suppression of B cells activation (Figure S2E). In vitro, butyrate attenuated OVA‐induced dendritic cell (DC) activation, reduced CD86 expression and restored IL‐10 production (Figures 1B, S2F,G), suggesting that SCFAs establish a tolerogenic environment for antigen presentation.

## ORAL NDF ALLEVIATES ESTABLISHED AR AND REPROGRAMS TH2‐DRIVEN ALLERGIC INFLAMMATION IN MICE

We next evaluated the therapeutic efficacy of NDF in an OVA‐induced AR mouse model [8] (Figure 1C). NDF was well tolerated without body‐weight loss (Figure S3A). Compared with allergic controls, NDF markedly reduced sneezing frequency, nasal OVA‐specific IgE, and nasal temperature responses after challenge (Figure 1D–F). NDF also increased IL‐10 while suppressing Th2‐associated cytokines, including IL‐4, IL‐5, and IL‐13, in nasal lavage fluid (Figures 1G, S3B). Systemically, NDF reduced serum OVA‐specific IgE levels (Figure S4A). Histological analysis further demonstrated reduced eosinophil infiltration and tissue edema in the nasal mucosa (Figure 1H), accompanied by a decreased proportion of GATA3^+^CD4^+^ Th2 cells (Figure S4B). In addition, NDF suppressed pro‐inflammatory cytokine production while increasing IL‐10 levels (Figure S4C,D). These results demonstrate that an oral NDF regimen markedly reduced OVA‐induced allergic symptoms in mice, while free OVA or OVA mixed with unencapsulated dextran had no effect, highlighting the requirement for the NDF formulation.

## NDF RESTORES INTESTINAL IMMUNE HOMEOSTASIS *VIA* SCFA‐FFAR2 SIGNALING

To elucidate the underlying mechanisms, we performed transcriptomic and high‐dimensional immunophenotypic analyses of intestinal tissues. RNA‐seq revealed that NDF treatment upregulated immune checkpoint genes (*lag3*, *pdcd1*, *ctla4*), *il10ra*, and other modulators (*cd40*, *cd40lg*, *cd5*, *timd2*) (Figures 1I, S5A), while Gene Ontology (GO) analysis indicated enrichment of biological processes related to immune regulation and B‐cell‐mediated immunity (Figures 1J, S5B–E). Mass cytometry (CyTOF) of intestinal immune cells further demonstrated that AR significantly expanded monocytes and macrophages, whereas NDF effectively reversed this shift and restored the abundance of T cells, B cells, and DCs, with the most prominent recovery observed in B cells (Figure S6). NDF‐treated mice also exhibited elevated expression of tolerogenic markers *pd‐1, foxp3*, and *cd103* in intestinal immune cell populations (Figure S7). Flow cytometry confirmed that NDF remarkably reduced intestinal Th2 cells (GATA3^+^CD4^+^) while increasing Tregs (FOXP3^+^CD4^+^), Bregs (CD1d^hi^CD5^+^), and tolerogenic DCs (CD103^+^CD11c^+^) (Figure S8). Collectively, these results indicate that oral NDF reprograms the intestinal immune landscape from a pro‐inflammatory Th2‐dominated state toward a regulatory phenotype.

To determine whether SCFA signaling is required for NDF‐mediated immune regulation, we administered NDF to *Ffar2*‐deficient mice (Figure S9A). In contrast to wild‐type mice, NDF failed to suppress Th2 cytokines or induce IL‐10 in nasal lavage fluid from *Ffar2*‐deficient mice (Figure S9B), and it also failed to expand intestinal Tregs and Bregs (Figure S9C,D). These findings establish that SCFA‐FFAR2 signaling is essential for the tolerogenic activity of NDF against AR, and support a mechanistic model in which microbial fermentation of dextran‐derived dietary fiber generates SCFAs that signal through FFAR2 to promote regulatory immune remodeling.

## GUT‐DERIVED BREGS MIGRATE TO THE NASAL MUCOSA AND MEDIATE LOCAL SUPPRESSION

**FIGURE 2:**
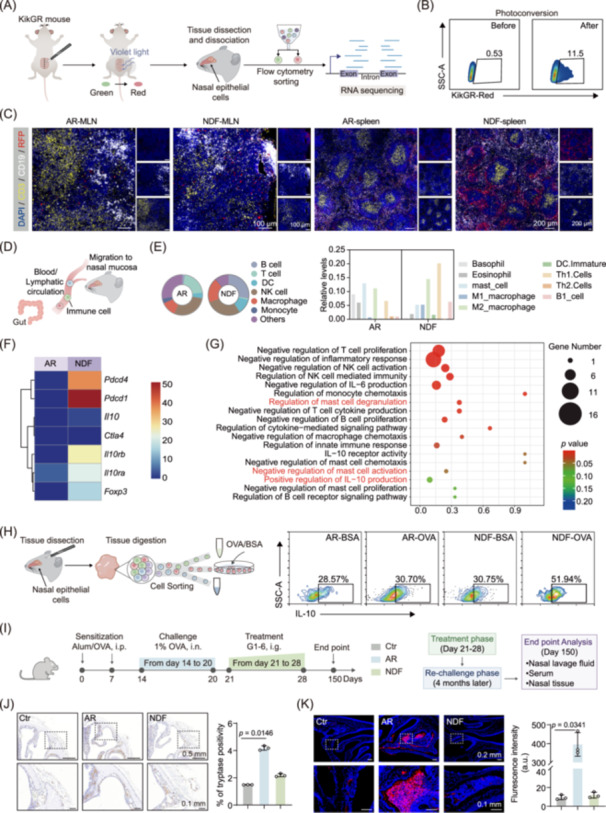
Gut‐derived immune cells migrate to the nasal mucosa and establish long‐term anti‐inflammatory immune memory following NDF treatment. (A) Schematic of the experimental strategy for tracking gut‐derived cells using KikGR transgenic mice. Following photoconversion of the KikGR protein from green to red fluorescence (405 nm illumination) in the gut, the migration of photoconverted (red) cells was tracked. Red fluorescent cells were isolated from dissociated nasal tissues by fluorescence‐activated cell sorting (FACS) for subsequent RNA‐seq analysis. (B) Flow cytometry analysis validating the efficiency of photoconversion in intestinal cells, comparing samples from non‐converted and photoconverted mice. (C) Representative immunofluorescence images of mesenteric lymph nodes (MLN) and spleen from AR and NDF‐treated groups after photoconversion. Tissues were stained for CD3 (T cells, yellow), CD19 (B cells, white), KikGR‐Red (photoconverted cells), and DAPI (nuclei, blue). Scale bars, 100 μm (MLN) and 200 μm (spleen). (D) The schematic highlights the gut‐to‐nose immune axis, illustrating that oral NDF induces gut‐trained immune cells that migrate to the nasal mucosa. (E) Immune cell composition analysis of sorted nasal KikGR‐Red^+^ cells. The relative abundance of major immune cell types (left) and detailed subsets (right) was inferred from RNA‐seq data using the ImmuCellAI algorithm. (F) Heatmap displaying RNA‐seq expression profiles of differentially expressed genes (DEGs) between KikGR‐Red^+^ cells from AR and NDF‐treated mice. (G) GO enrichment analysis (bubble plot) of biological processes related to immune regulation that are upregulated in NDF‐derived KikGR‐Red^+^ cells. (H) Cytokine production by sorted nasal KikGR‐Red^+^ cells after ex vivo restimulation with OVA or BSA (control) for 3 h. The percentage of IL‐10^+^ cells was analyzed by flow cytometry. (I) Schematic timeline of allergic rhinitis induction and treatment. Four months after the final treatment, mice were re‐challenged, and samples, including nasal lavage fluid, serum for cytokine analysis, and nasal tissues, were collected to assess long‐term anti‐inflammatory immune memory. (J) Representative immunohistochemical staining of mast cell tryptase in nasal tissues after treatment, with enlarged views (scale bars: upper, 0.5 mm; lower, 0.1 mm). Tryptase‐positive area quantification is shown on the right. (K) Representative immunofluorescence staining of GATA3 in nasal tissues to assess Th2 cells after treatment, with enlarged views (scale bars: upper, 0.2 mm; lower, 0.1 mm). Quantification of GATA3 fluorescence intensity is shown on the right. Data in (J) and (K) are presented as mean ± SD (*n* = 3). Statistical analysis was performed using one‐way ANOVA followed by Tukey's post‐hoc test.

We next investigated whether NDF‐induced intestinal regulatory cells could migrate to the nasal mucosa to suppress local inflammation. To track cell trafficking in vivo, we employed KikGR photoconvertible mice, in which cells can be irreversibly labeled by violet light (405 nm) from green to red fluorescence (Figure 2A). Following intestinal photoconversion, we observed a significant increase in red fluorescent protein‐positive (RFP^+^) cells in the nasal mucosa of NDF‐treated mice (~11%) (Figure 2B). Immunofluorescence staining revealed RFP^+^ cells co‐localizing with B and T cells in Peyer's patches, while in the spleen, RFP^+^ cells from NDF‐treated mice showed a stronger association with B cells (Figure 2C). Nasal mucosal RFP^+^ cells were significantly increased after NDF treatment (Figure S10A), indicating enhanced gut‐to‐nose trafficking (Figure 2D). RNA‐seq of sorted RFP^+^ nasal cells, followed by ImmuCellAI analysis, revealed that NDF increased the proportions of B cells, DCs, and macrophages, and decreased T cells and monocytes (Figure 2E). Subpopulation analysis indicated reductions in basophils, eosinophils, mast cells, and Th2 cells, alongside increases in B1 cells, Th1 cells, and M1/M2 macrophages (Figure 2E). The migrated cells also upregulated *il10ra*, *il10rb*, *ctla4*, *pdcd1*, and *foxp3* (Figure 2F), consistent with a regulatory transcriptional program. GO analysis further highlighted enrichment of negative regulation of immune cell chemotaxis, inhibition of mast cell activation, and promotion of IL‐10 signaling (Figure 2G), suggesting a regulatory transcriptional program in migrated cells. To functionally characterize these migrated cells, we isolated RFP^+^ nasal cells and restimulated them with OVA ex vivo. Cells from NDF‐derived mice produced significantly more IL‐10 compared with controls (Figure 2H). Strikingly, 73.4% of IL‐10^+^ cells were CD19^+^ B cells, while 25.6% were CD3^+^ T cells (Figure S10B). These data identify IL‐10‐producing Bregs as the major gut‐origin populations that migrate to the nasal mucosa after NDF treatment and contribute to antigen‐specific tolerance.

## NDF INDUCES DURABLE PROTECTION AGAINST ALLERGIC RELAPSE

To evaluate the durability of NDF‐induced tolerance against allergic relapse, we rechallenged mice with intranasal OVA 4 months after completing the 8‐day NDF treatment course (Figure 2I). Remarkably, NDF‐treated mice maintained nasal IL‐4, histamine, and OVA‐specific IgE levels comparable to healthy controls, while IL‐10 remained elevated (Figure S11A). Other treatments, including the clinical antihistamine desloratadine, provided only partial protection. Serum cytokine levels at 15 min post‐challenge did not differ significantly among groups (Figure S11B), possibly due to the short sampling time. Histology revealed reduced mast cell tryptase expression and decreased GATA3^+^ Th2 cells in nasal tissue in the NDF group (Figures 2J,K, S11C,D), further supporting the evidence of sustained immune modulation. These findings indicate that a short‐course oral NDF regimen does not merely suppress acute symptoms, but also induces a potential long‐term protective effect against allergic recurrence for at least 4 months.

## ORAL NDF CONFERS BROAD PROTECTION AGAINST CLINICALLY RELEVANT POLLEN ALLERGEN

We further validated the generalizability of the NDF platform using mugwort pollen extract, a clinically relevant aeroallergen (Figure S12A). In a mugwort pollen‐induced AR mouse model [20], pollen‐loaded NDF (pNDF) significantly increased CD1d^hi^CD5^+^ Bregs and FOXP3^+^ Tregs in mesenteric lymph nodes and Peyer's patches, with similar expansions observed in the spleen, accompanied by reduced Th2 cells and increased tolerogenic DCs (Figures S12B,C, S13A). Functionally, pNDF‐treated mice showed normal perinasal appearance, reduced sneezing, and increased IL‐10^+^ B cells in nasal mucosa (Figure S13B–D). Histological analysis confirmed reduced epithelial inflammation and eosinophil infiltration (Figure S13E).

Given the close connection between AR and asthma, we further examined whether oral NDF could alleviate lower‐airway allergic disease (Figure S14A). In an OVA‐induced asthma model [8], NDF improved pulmonary function, with peak inspiratory flow and enhanced pause returning toward healthy levels (Figure S14B,C), and reduced serum OVA‐specific IgE after challenge without affecting body weight (Figure S14D,E). Collectively, these results demonstrate that oral NDF provides broad protection against both upper and lower airway allergic diseases.

In summary, we have developed an oral NDF platform that functions as a therapeutic tolerance‐inducing vaccine for allergic airway diseases. This finding challenges the traditional T‐cell‐centric view of oral tolerance and positions Bregs as primary cellular executors through the gut‐nose axis. We demonstrate that this food‐derived, fermentable nanomaterial induces oral tolerance against aeroallergens by promoting the migration of gut‐resident IL‐10^+^ Bregs to the nasal mucosa through the SCFA‐FFAR2 axis. Mechanistically, NDF combines sustained allergen delivery with in situ production of immunoregulatory metabolites, linking antigen‐specific and microbiota‐mediated tolerance pathways. Conceptually, NDF co‐delivers an allergen and an immunomodulatory adjuvant, analogous to vaccine design, but instead drives antigen‐specific immune tolerance rather than protective immunity. This strategy integrates dietary biomaterials with immune modulation and may provide a safe, patient‐friendly alternative to current immunotherapies. Given its modular design, NDF could be adapted to different allergens and potentially developed as a personalized therapy for polysensitized patients. Although further studies are needed to optimize dosing, evaluate long‐term safety, and characterize the microbial taxa responsible for NDF fermentation, these findings establish a strong preclinical foundation for harnessing the gut‐nose axis to prevent and treat allergic airway diseases.

## AUTHOR CONTRIBUTIONS

**Yuting Qin**: Methodology; software; data curation; formal analysis; validation; visualization; resources; investigation; writing—original draft. **Zeming Wang**: Methodology; validation; visualization; investigation; software; formal analysis; data curation; resources; writing—original draft. **Yuru Zong**: Methodology; software; data curation; validation; formal analysis. **Zefang Lu**: Methodology; validation. **Junxiu Liu**: Conceptualization; supervision; project administration. **Ruifang Zhao**: Conceptualization; funding acquisition; project administration; supervision. **Guangjun Nie**: Conceptualization; funding acquisition; writing—review and editing; project administration; supervision. **Hanqing Chen**: Conceptualization; funding acquisition; writing—review and editing; writing—original draft; project administration; supervision; methodology.

## CONFLICT OF INTEREST STATEMENT

The authors declare no conflicts of interest.

## ETHICS STATEMENT

All animal experiments were conducted in accordance with the ethical guidelines for animal research and received approval from the Ethics Committee of the National Center for Nanoscience and Technology (No. NCNST21‐202511‐0140). In this study, we rigorously adhered to protocols for monitoring animal welfare and performing euthanasia.

## Supporting information


**Table S1:** Univariable logistic regression analysis results.
**Table S2:** NHANES survey‐weighted logistic regression.
**Table S3:** Multivariable logistic regression analysis results.
**Table S4:** Association between dietary fiber intake and AR using two modeling approaches (adjusted for age and gender).

**Figure S1:** Population‐based evidence of a linear inverse association between dietary fiber intake and AR.
**Figure S2:** Design of a nano‐dietary fiber (NDF)‑based oral antigen delivery system.
**Figure S3:** NDF treatment is well tolerated and suppresses Th2‐associated inflammation.
**Figure S4:** NDF suppresses local Th2 responses and systemic allergic inflammation.
**Figure S5:** NDF promotes immunoregulatory gene expression and B cell–associated pathways in the intestine.
**Figure S6:** CyTOF profiling of intestinal lymphocyte subsets at endpoint.
**Figure S7:** Increased expression of tolerogenic markers in intestinal immune cells following NDF treatment.
**Figure S8:** NDF reprograms the intestinal immune landscape toward a regulatory phenotype.
**Figure S9:** Comparison of allergic rhinitis induction in *Ffar2* ^−/−^ mice.
**Figure S10:** Gut‐derived B cells constitute the major IL‐10‐producing population in the nasal mucosa.
**Figure S11:** Cytokine profiles and cellular analysis in nasal tissues post‐allergen challenge.
**Figure S12:** Immunomodulatory and therapeutic effects in murine models of mugwort‐induced rhinitis.
**Figure S13:** pNDF promotes regulatory immune responses and alleviates mugwort pollen‐induced allergic rhinitis.
**Figure S14:** NDF alleviates allergic asthma and improves pulmonary function.

## Data Availability

The RNA sequencing data generated in this study have been deposited in the NCBI Sequence Read Archive (SRA) under BioProject accession numbers PRJNA1371643 (immune cell migration from gut to nasal mucosa, https://www.ncbi.nlm.nih.gov/bioproject/PRJNA1371643) and PRJNA1372444 (intestinal transcriptome of AR mice and NDF‐treated mice, https://www.ncbi.nlm.nih.gov/bioproject/PRJNA1372444). All data necessary for evaluating the conclusions presented in this paper are included within the paper or in the supplementary information. The data and scripts used are saved in GitHub (https://github.com/qinyt-hub/mimic-allergy). Supplementary materials (methods, figures, tables, graphical abstract, slides, videos, Chinese translated version, and updated materials) may be found in the online DOI or iMeta Science http://www.imeta.science/. The data that support the findings of this study are available from the corresponding author upon reasonable request.
