# Supplementary material for: Dietary fiber‐based oral nanovaccine promotes gut‐to‐nose trafficking of regulatory cells and drives antigen‐specific tolerance to relieve airway allergy
Source: Imeta. 2026 Jul 25:e70158. Online ahead of print. doi: 10.1002/imt2.70158 (PMC13401610; doi:10.1002/imt2.70158)
Supplement: Supplementary file 2 — Figure S1: Population‐based evidence of a linear inverse association between dietary fiber intake and AR. Figure S2: Design of a nano‐dietary fiber (NDF)‑based oral antigen delivery system. Figure S3: NDF treatment is well tolerated and suppresses Th2‐associated inflammation. Figure S4: NDF suppresses local Th2 responses and systemic allergic inflammation. Figure S5: NDF promotes immunoregulatory gene expression and B cell–associated pathways in the intestine. Figure S6: CyTOF profiling of intestinal lymphocyte subsets at endpoint. Figure S7: Increased expression of tolerogenic markers in intestinal immune cells following NDF treatment. Figure S8: NDF reprograms the intestinal immune landscape toward a regulatory phenotype. Figure S9: Comparison of allergic rhinitis induction in Ffar2 −/− mice. Figure S10: Gut‐derived B cells constitute the major IL‐10‐producing population in the nasal mucosa. Figure S11: Cytokine profiles and cellular analysis in nasal tissues post‐allergen challenge. Figure S12: Immunomodulatory and therapeutic effects in murine models of mugwort‐induced rhinitis. Figure S13: pNDF promotes regulatory immune responses and alleviates mugwort pollen‐induced allergic rhinitis. Figure S14: NDF alleviates allergic asthma and improves pulmonary function. [file IMT2-9999-e70158-s001.docx]

**Supporting information to**

**Dietary fiber-based oral nanovaccine promotes gut-nose trafficking of regulatory cells and drives antigen-specific tolerance to relieve airway allergy**

**Running title**: Fiber-based oral nanovaccine induces gut–nose immune tolerance

Yuting Qin^1,2#^, Zeming Wang^1,3#^, Yuru Zong^4^, Zefang Lu^1^, Junxiu Liu^5^*, Ruifang Zhao^1,3^*, Guangjun Nie^1,3^*, Hanqing Chen^4^*

^1^ Beijing Key Laboratory for Drug Delivery Nanocarriers, CAS Center for Excellence in Nanoscience, National Center for Nanoscience and Technology, Beijing 100190, China

^2^ Institute of Nanotechnology And Intelligence (inAI), Jinan University, Guangzhou 510632, China

^3^ Center of Materials Science and Optoelectronics Engineering, University of Chinese Academy of Sciences, Beijing 100049, China

^4^ Beijing Key Laboratory of Environment and Aging, Department of Nutrition and Food Hygiene, School of Public Health, Capital Medical University, Beijing 100069, China

^5^ Department of Otolaryngology-Head and Neck Surgery, Peking University First Hospital, Beijing 100034, China

^#^ These authors contributed equally: Yuting Qin, Zeming Wang

*Correspondence: [junxiuliu@bjmu.edu.cn](mailto:junxiuliu@bjmu.edu.cn) (Junxiu Liu); [zhaorf@nanoctr.cn](mailto:zhaorf@nanoctr.cn) (Ruifang Zhao);

[niegj@nanoctr.cn](mailto:niegj@nanoctr.cn) (Guangjun Nie); [chenhq@ccmu.edu.cn](mailto:chenhq@ccmu.edu.cn) (Hanqing Chen)

**SUPPLEMENTARY METHODS**

**Study population and analysis**

Data were obtained from the National Health and Nutrition Examination Survey (NHANES), a nationally representative cross-sectional study conducted by the National Center for Health Statistics (NCHS). The analysis used data from the NHANES 2017–March 2020 pre-pandemic cycle. The study protocol was approved by the NCHS Institutional Review Board. Using four years of NHANES data with available information on dietary fiber intake and allergic rhinitis (AR), we excluded participants with missing dietary fiber data, missing AR diagnosis, or incomplete covariates (age, sex, race/ethnicity, body mass index (BMI), smoking status, poverty income ratio (PIR), and total energy intake). We incorporated Mobile Examination Center (MEC) examination weights (WTMECPRP), stratification variables (SDMVSTRA), and primary sampling units (SDMVPSU) using a survey-weighted generalized linear model framework. The final sample comprised 8,092 adults aged 18–80 years (3,950 men and 4,142 women). The data were analyzed sequentially, including descriptive statistics, univariable and multivariable logistic regression, continuous exposure modeling, and trend tests across ordered categories. Restricted cubic spline (RCS) analysis was used to examine potential nonlinearity in the association between fiber intake and AR risk.

**Synthesis and characterization of dextran nanoparticles**

Ovalbumin (Grade V, Sigma-Aldrich) was encapsulated within NDF using an aqueous-aqueous emulsion method based on freezing-induced phase separation. Specifically, the protein/pollen was initially dissolved in a 6% (w/w) dextran solution (molecular weight 40 kDa, YEASEN), while polyethylene glycol 2000 (PEG2000, Sinopharm) was prepared separately as a 6% (w/v) aqueous solution. The two solutions were then combined under gentle stirring to form a homogeneous mixture. This mixture was subsequently frozen at –80 °C and lyophilized under vacuum for 24 hours. The resulting powder underwent repeated washing with dichloromethane and centrifugation at 12,000 rpm to remove residual PEG. Following complete solvent evaporation, well-dispersed nano-dextran encapsulating ovalbumin were successfully obtained. Morphological characterization was conducted using a scanning electron microscope (SU-8010, Hitachi). To assess formulation efficiency, NDFs were synthesized at varying dextran-to-ovalbumin mass ratios (1:0, 4:1, 2:1, and 1:1). The encapsulation efficiency was indirectly determined by quantifying the unencapsulated protein present in the supernatant after centrifugation. The NDF suspension was subjected to centrifugation at 12,000 rpm for 10 minutes, after which the supernatant was analyzed using a Bradford protein assay kit (Bio-Rad, UK). A standard curve was generated according to the manufacturer's protocol. The corrected optical density values were employed to calculate free ovalbumin concentration and derive encapsulation parameters.

**Imaging intestinal retention**

Rhodamine-labeled OVA was synthesized by conjugating NHS-rhodamine (Thermo Scientific) to OVA through amidation at room temperature, resulting in a stable covalent bond. To produce fluorescently labeled NDF, OVA, and rhodamine were co-dissolved in a 6% dextran solution, mixed with a 6% PEG solution, and subsequently lyophilized. The obtained powder was washed to yield rhodamine-loaded NDF.

Alum/OVA-sensitized BALB/c mice were orally administered 150 μL of either free rhodamine-OVA or rhodamine-encapsulated NDF. Following gavage, food was withheld from the mice, which were euthanized at time points of 0.5, 4, 8, 12, and 16 h post-administration. The gastrointestinal tract was harvested and imaged using an IVIS optical imaging system to assess intestinal retention and metabolism.

**Establishment of murine models for allergic airway diseases**

All animal studies were conducted in accordance with protocols approved by the Ethics Committee of the National Center for Nanoscience and Technology. Female BALB/c mice (6 weeks old) were obtained from SPF (Beijing) Biotechnology Co., Ltd, and housed under standard conditions. After 7 days of acclimation, mice were co-housed throughout the sensitization phase.

To establish the OVA-induced AR model, BALB/c mice were administered intraperitoneal injections of 75 μg OVA with 2 mg alum in 0.2 mL PBS on days 0 and 7. A naive control group received PBS alone. From days 14 to 20, sensitized mice were challenged daily via intranasal instillation of 20 μL PBS containing 1% OVA, while controls received PBS. On day 21, sensitized mice were randomized into treatment groups and administered PBS, OVA, OVA plus dextran, or NDF by oral gavage for 8 days. The OVA dose was escalated from 0.5 mg to 3.5 mg, with dextran maintained at 4 mg per mouse. An additional AR model was generated using *Ffar2*-knockout C57BL/6N mice, provided by Professor Hanqing Chen (Capital Medical University), following the same sensitization and challenge protocol.

Mugwort pollen-induced allergic rhinitis was established in mice through systemic sensitization followed by local airway challenge. Sensitized mice received two intraperitoneal injections of mugwort pollen extract (Stallergenes Greer), one week apart (days 0 and 7), each containing 1000 PNU in 200 µL PBS absorbed to 2 mg aluminum hydroxide. Negative controls received 200 µL PBS with alum. From day 14, all mice underwent daily intranasal challenge for 7 days: sensitized mice received 200 PNU pollen extract in 20 µL PBS, while controls received 20 µL PBS alone. Therapeutic oral gavage was administered from day 21 to 28. Sensitized mice were divided into groups receiving PBS, free pollen extract, pollen extract with dextran, or pNDF. The oral pollen dose was escalated from 200 to 3000 PNU per mouse, with dextran maintained at 4 mg per mouse.

In parallel, an OVA-induced asthma model was generated. Mice were sensitized by intraperitoneal injection of 75 µg OVA and 2 mg aluminum hydroxide in 200 µL PBS on days 0 and 7; controls received 200 µL PBS. From days 14 to 20, sensitized mice were exposed daily to a 3% (w/v) OVA aerosol for 15 minutes via jet nebulizer.

Body weight was monitored throughout the study. In the AR models, allergic symptoms were evaluated by recording the number of sneezing episodes during a 15-min period following the final allergen challenge. Airway function in asthmatic mice was assessed using unrestrained whole-body plethysmography. At the experimental endpoint, blood samples were collected for the determination of total and allergen-specific IgE levels. Small intestinal luminal contents were harvested for short-chain fatty acid (SCFA) analysis, while small intestinal tissues were collected for transcriptomic profiling. Cytokine levels were subsequently quantified, and histopathological examinations were performed to assess allergic inflammation in relevant tissues. RNA sequencing data were analyzed using the Majorbio Cloud Platform (https://cloud.majorbio.com) following the standard bioinformatics workflow provided by the platform [1]. Raw reads were subjected to quality control, including adapter trimming and removal of low-quality reads (Q < 20). Clean reads were aligned to the reference genome using a standard aligner (e.g., HISAT2), and gene-level counts were generated for downstream analysis. Differential expression analysis was performed using DESeq2 based on a negative binomial model, with normalization of read counts and adjustment for multiple testing using the Benjamini–Hochberg method. Genes with an adjusted *p* value (FDR) < 0.05 and |log2 fold change| > 1 were considered significantly differentially expressed.

**Histological analysis of the nasal mucosa**

The nasal mucosa was exposed by removing the lower jaw and dissecting away the surrounding skin, muscle, and soft tissues from the nasal region. The nasal tissue was fixed in 4% paraformaldehyde, decalcified in EDTA solution for one week, and then embedded in paraffin for sectioning. Tissue sections were stained with hematoxylin and eosin (H&E) and Giemsa stain to evaluate inflammatory lesions, eosinophil infiltration, and structural changes in the nasal epithelia.

**Multiplex cytokine/chemokine assay**

Blood was collected from mice into anticoagulant tubes and allowed to clot at room temperature for 1 hour. After centrifugation at 3000 rpm for 10 minutes, the supernatant serum was collected and stored at -80°C for subsequent cytokine analysis.

For nasal lavage fluid (NALF) collection, nasal tissues were exposed, and the nasal cavity was gently flushed with 1 mL of ice-cold PBS using a syringe. The effluent was collected from the nostrils and immediately stored at -80°C.

Prior to the multiplex assay, NALF samples were thawed, and total protein concentration was determined using a BCA assay. All samples were adjusted to a uniform protein concentration with PBS for normalization. Cytokine and chemokine levels were simultaneously measured using a Luminex-based multiplex assay (R&D Systems) according to the manufacturer's protocol. Results were normalized to the total protein content in each sample.

**Measurement of OVA-specific IgE antibodies**

Serum and NALF were collected from mice, and levels of OVA-specific IgE were measured using enzyme-linked immunosorbent assay (ELISA). An indirect ELISA procedure was employed to quantify OVA-specific IgE in serum, in accordance with the manufacturer’s instructions for the mouse IgE ELISA kit (Gelatins Biological Reagent Co., Ltd). Results are expressed in μg/mL.

***In Vivo* tracking of immune cell migration from gut to nasal mucosa**

KikGR transgenic mice (Catalog #I001211, Cyagen) were used to establish an OVA-induced allergic rhinitis model via systemic sensitization followed by intranasal challenge with 1% OVA. After treatment with NDF, sensitized mice underwent in vivo photoconversion. Mice were anesthetized with tribromoethanol, and a midline abdominal incision (~1 cm) was made to expose the small intestine. The mouse body was covered with sterile aluminum foil, leaving only the intestinal region exposed. The small intestine was irradiated with 405 nm ultraviolet light at 800 mW/cm² for 8 minutes, then carefully returned to the abdominal cavity. The incision was sutured and disinfected. After 24 hours, the mice were euthanized, and both intestinal and nasal mucosal tissues were collected for subsequent flow cytometric analysis and RNA sequencing.

**Isolation and analysis of nasal mucosal cells**

Nasal mucosa tissues were enzymatically dissociated in HBSS buffer containing 0.1 mg/ml collagenase IV (Gibco) and 0.05 mg/ml DNase I (Solarbio), and then incubated at 37°C with shaking at 100 rpm for 40 minutes. The resulting cell suspension was filtered through a 70 μm strainer and centrifuged at 400 × g for 5 minutes at 4°C. After removing the supernatant, red blood cell lysis buffer was added, followed by another centrifugation step under the same conditions. The isolated nasal mucosal cells were then resuspended in PBS containing 2% FBS.

Cells were sorted using a CytoFLEX SRT flow cytometer (Beckman). KikGR-Red positive populations were gated and collected based on 594 nm fluorescence. A subset of sorted cells was used for RNA-seq. These cells were preserved in RNAlater (Thermo Fisher), and total RNA was extracted with Trizol reagent (Invitrogen) according to the manufacturer’s protocol. RNA concentration and integrity were assessed using a NanoDrop ND-1000 spectrophotometer and an Agilent Bioanalyzer 2100, respectively. RNA sequencing and bioinformatic analysis were performed by LC-Bio Technology Co., Ltd. (Zhejiang, China).

Another portion of KikGR-Red positive cells was stimulated in vitro with 1% OVA or BSA at 37°C for 6 hours, followed by a 2-hour treatment with brefeldin A to inhibit cytokine secretion. For intracellular cytokine staining, cells were first blocked with anti-CD16/32 and then stained with the following antibody panel: APC/Cyanine7 anti-mouse CD45 (clone QA17A26, Biolegend), Pacific Blue anti-mouse CD3 (clone 17A2, Biolegend), PerCP anti-mouse CD4 (clone GK1.5, Biolegend), Brilliant Violet 605 anti-mouse CD19 (clone 1D3/CD19, Biolegend), and FITC anti-mouse IL-10 (clone JES5-16E3, Biolegend). Flow cytometry data were acquired on a CytoFLEX instrument (Beckman).

**Mass cytometry for immune cell staining and analysis**

Gut cell processing and staining were conducted according to a standardized protocol. In brief, cells were initially stained for viability using cisplatin, followed by incubation with a panel of metal-conjugated surface antibodies. After surface staining, the cells were fixed and permeabilized; intracellular staining was performed where applicable. Nucleic acids were labeled with an iridium intercalator, after which the cells were washed and resuspended in Milli-Q water containing normalization beads. Data acquisition was executed on a Helios/Fluidigm mass cytometer. Raw data underwent normalization utilizing bead-based algorithms (Normalizer v0.3) to ensure consistency across experiments. For immune cell analysis, a sequential gating strategy was employed: intact cells were first gated based on iridium DNA staining, followed by the selection of live single cells through event length assessment and exclusion of cisplatin. Dimensionality reduction was achieved via viSNE analysis in Cytobank, incorporating lineage markers such as B220, CD3, CD4, CD8, CD11b, CD11c, CD19, and CD45 for population discrimination. Cell clusters were manually annotated based on t-SNE projections and the expression patterns of specific markers. Hierarchical clustering was performed using Cluster 3.0 with average linkage; results were visualized employing Java TreeView and GraphPad. All mass cytometry experiments were carried out at Zhejiang PLT Technology Co., Ltd.

**Immunophenotypic analysis of small intestine and mesenteric lymph nodes**

Mesenteric lymph nodes (MLNs), Peyer’s patches, and small intestinal tissues were collected for flow cytometric analysis. For MLNs and Peyer’s patches, tissues were mechanically dissociated through a 70 μm filter and resuspended in PBS containing 2% FBS. For small intestinal samples, tissues were cut into small fragments, thoroughly washed, and incubated in HBSS containing 1 mM DTT, 2.5% FBS, and 1 mM EDTA at 37 °C for 30 minutes with agitation. The fragments were then digested enzymatically in HBSS supplemented with 1% collagenase IV and 0.1% DNase I at 37 °C for 60 minutes. The resulting cell suspension was separated using a discontinuous Percoll gradient (40%/75%), and cells at the interface were collected for further analysis. All cells were pre-blocked with anti-CD16/32 and stained with the following antibody panel: Brilliant Violet 605^TM^ anti-mouse CD45 (clone S18009F), FITC anti-mouse CD45 (clone S18009F), PerCP/Cyanine5.5 anti-mouse CD45 (clone S18009F), PE anti-mouse CD1d (clone K253), Alexa Fluor® 488 anti-mouse CD19 (clone 6D5), APC anti-mouse CD5 (clone 53-7.3), Brilliant Violet 421^TM^ anti-mouse CD11c (clone N418), PE/Cyanine7 anti-mouse CD103 (clone 2E7), APC/Cyanine7 anti-mouse CD4 (clone GK1.5), Alexa Fluor® 488 anti-GATA3 (clone 16E10A23), Pacific Blue^TM^ anti-FOXP3 (clone MF-14), PE anti-mouse/human CD11b (clone M1/70), Brilliant Violet 605^TM^ anti-mouse F4/80 (clone BM8), and APC anti-mouse CD86 (clone A17199A), all from BioLegend. Data acquisition was performed on a CytoFLEX flow cytometer (Beckman).

**Statistical analyses, reproducibility and figure preparation**

Statistical analyses were performed using GraphPad Prism v.10 and R (RStudio). For NHANES data, associations between dietary fiber intake and allergic rhinitis were evaluated using univariable and multivariable logistic regression models, and effect estimates were reported as odds ratios with 95% confidence intervals. Categorical variables were assessed using chi-square tests. Survey-weighted regression outputs were additionally visualized using forest plots generated in R. For group comparisons, unpaired two-tailed Student’s t-tests were applied to normally distributed data, whereas non-parametric Mann–Whitney U tests were used when normality assumptions were not met. For comparisons involving more than two groups, one-way analysis of variance (ANOVA) was used, followed by Tukey’s post-hoc test for multiple pairwise comparisons, or Dunnett’s test when comparisons were made against a single control group. Repeated or longitudinal measurements were analyzed using appropriate models accounting for within-subject variation, as specified in the corresponding figure legends. Categorical variables were analyzed using chi-square tests where applicable. All statistical tests were two-sided, and statistical significance was defined as *p* < 0.05. For animal experiments, each biological replicate corresponded to an independent animal, and replicate numbers are indicated in the figure legends. In all cases, technical replicates were averaged prior to statistical analysis. Figures were generated using GraphPad Prism 8.0.2, R 4.6.1, ImmuCellAI 2.0 and Adobe Illustrator v.29.0, and were assembled for clarity and consistency across panels.

**Reference**

1. Zhao, Jianhua, Linmeng Liu, Jichen Han, Junbiao Zhang, Xiaodan Li, Yan Wang, Qianhui Han, et al. 2026. “Majorbio Cloud 2026 provides comprehensive analysis workflows for microbiome.” *iMeta* 5: e70109. <https://doi.org/https://doi.org/10.1002/imt2.70109>

**SUPPLEMENTARY FIGURES**


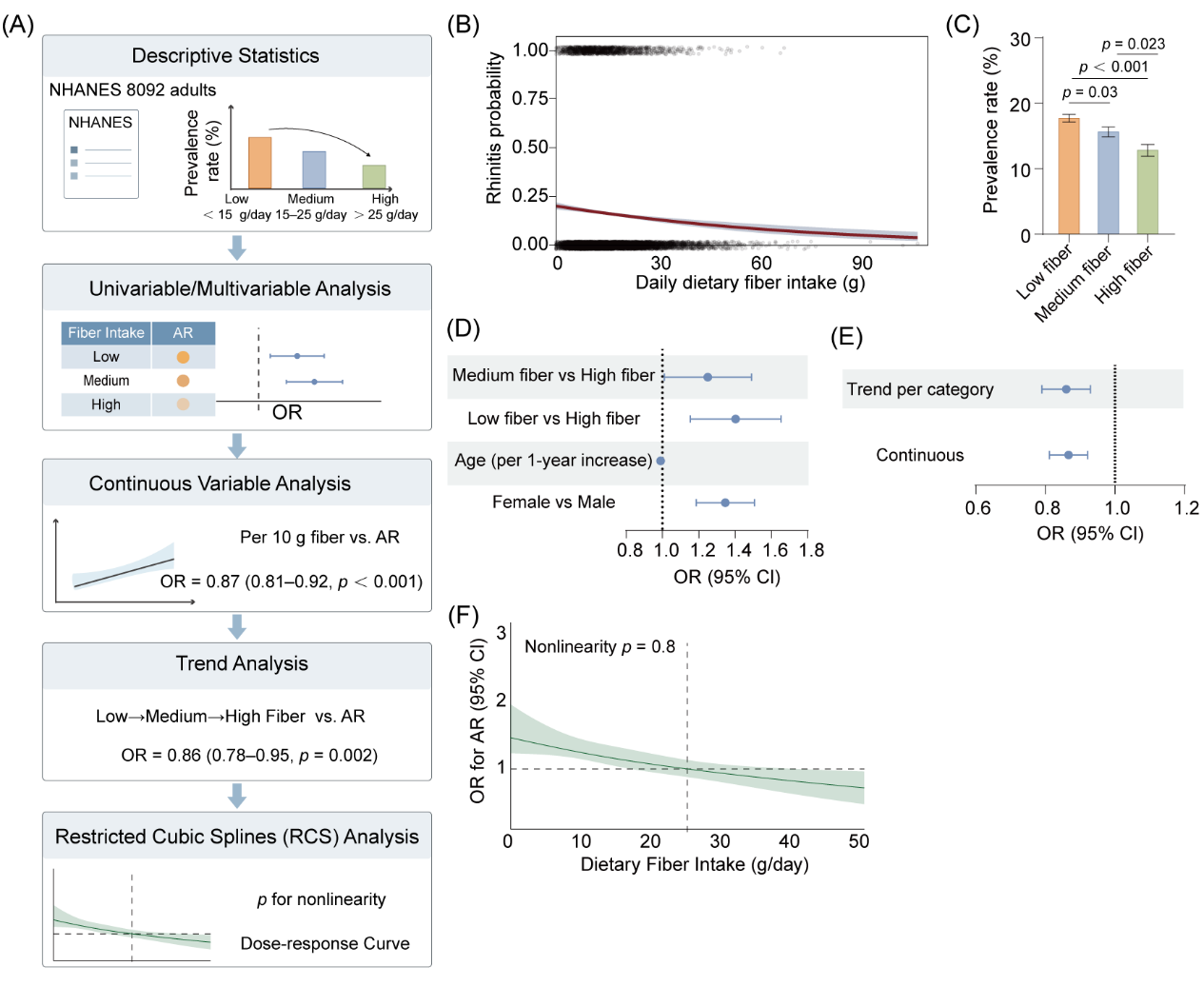


**Figure S1 Population-based evidence of a linear inverse association between dietary fiber intake and AR. (A)** Analytical workflow for assessing the association between dietary fiber intake and AR. Analyses were conducted sequentially using descriptive statistics, followed by univariable and multivariable logistic regression, continuous exposure modeling, and trend testing across ordered categories. Restricted cubic spline (RCS) analysis was applied to evaluate potential nonlinearity in the association between dietary fiber intake and AR risk. **(B) Predicted probability of AR by dietary fiber intake. Data were from the U.S. National Health and Nutrition Examination Survey (NHANES), including 8,092 participants aged 18**–**80 years (3,950 men and 4,142 women). (C) Prevalence of AR across different fiber intake groups: High Fiber ( > 25 g/day), Medium Fiber (15**–**25 g/day), and Low Fiber ( < 15 g/day). *p*-values were derived from the chi-square goodness-of-fit test. (D)** **Forest plot showing the results of multivariable logistic regression analysis. (E)** **Forest plot of the association between dietary fiber intake and AR. Intake was analyzed as a continuous variable (per 10 g day⁻¹ increase) and as categorical groups ( < 15, 15**–**25, > 25 g /day) using multivariable logistic regression adjusted for age and sex. (F) RCS analysis showing the association between dietary fiber intake and AR risk, adjusted for age and sex. The reference value was 25 g/day. The dose–response relationship was linear (*p* for nonlinearity = 0.80).**


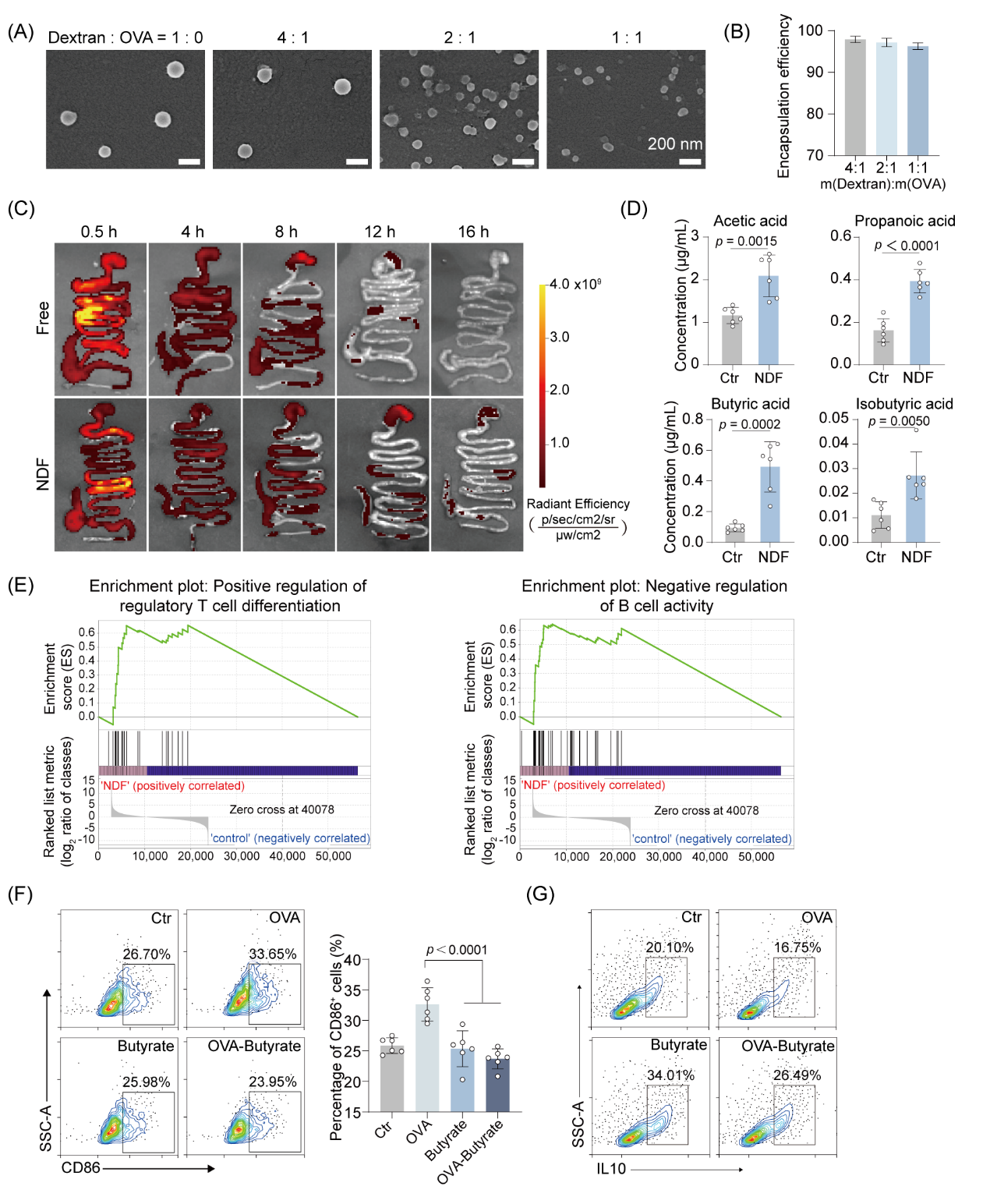


**Figure S2 Design of a nano dietary fiber (NDF)‑based oral antigen delivery system. (A)** SEM micrographs of NDF synthesized with varying mass ratios of dextran to OVA. Scale bar, 200 nm. **(B)** **Encapsulation efficiency of dextran nanoparticles at varying dextran-to-OVA mass ratios (4:1, 2:1, 1:1). (C)** **Representative in vivo IVIS images at indicated times (0.5, 4, 8, 12, and 16 h) post-administration of free OVA or NDF-encapsulated OVA. (D)** **Quantification of SCFAs (acetate, propionate, butyrate, and isobutyrate) in intestinal contents after oral NDF treatment for 3 days. (E)** **GSEA of intestinal tissues from NDF versus control groups. GSEA plot showing significant enrichment of the pathway “positive regulation of regulatory T cell differentiation” (left); GSEA plot showing significant enrichment of the pathway “negative regulation of B cell activity” (right). (F) Flow cytometric analysis of CD86^+^ DCs after overnight incubation with OVA, butyrate, or OVA + butyrate. Representative plots and summary bar graph are shown. (G)** **Representative flow cytometry plots of IL-10^+^ dendritic cells following stimulation.**


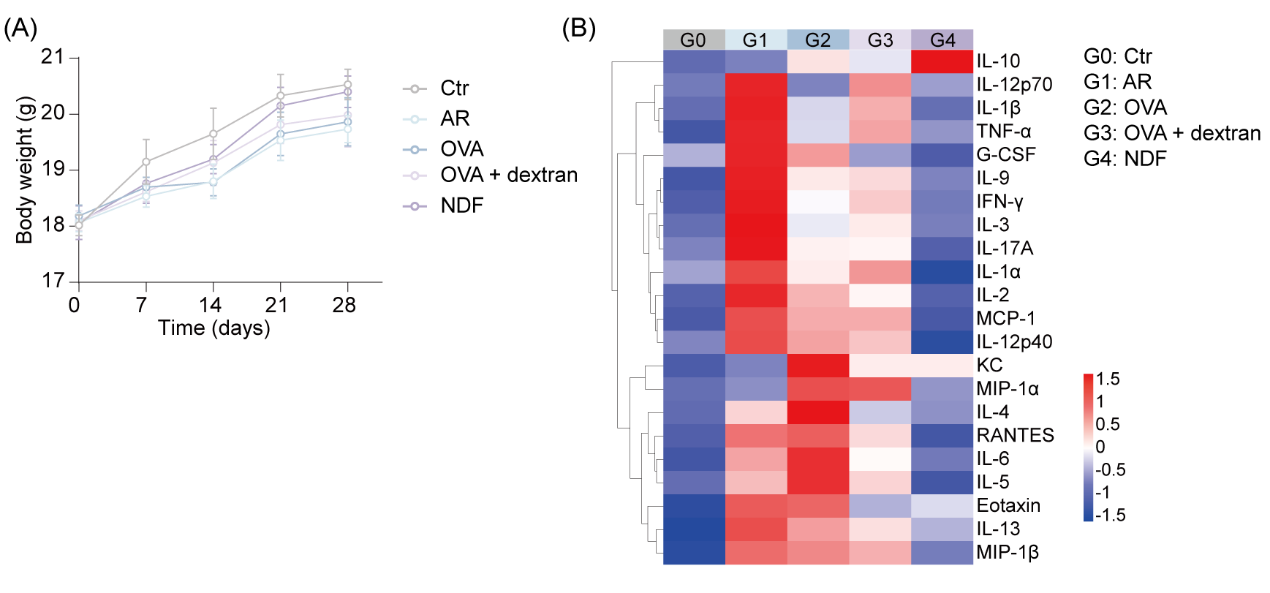


**Figure S3 NDF treatment is well tolerated and suppresses Th2-associated inflammation. (A)** Monitoring body weight in mice with rhinitis during therapeutic intervention. **(B) Heatmap depicting the relative levels of Th2-associated cytokines and the immunoregulatory cytokines in nasal lavage fluid.**


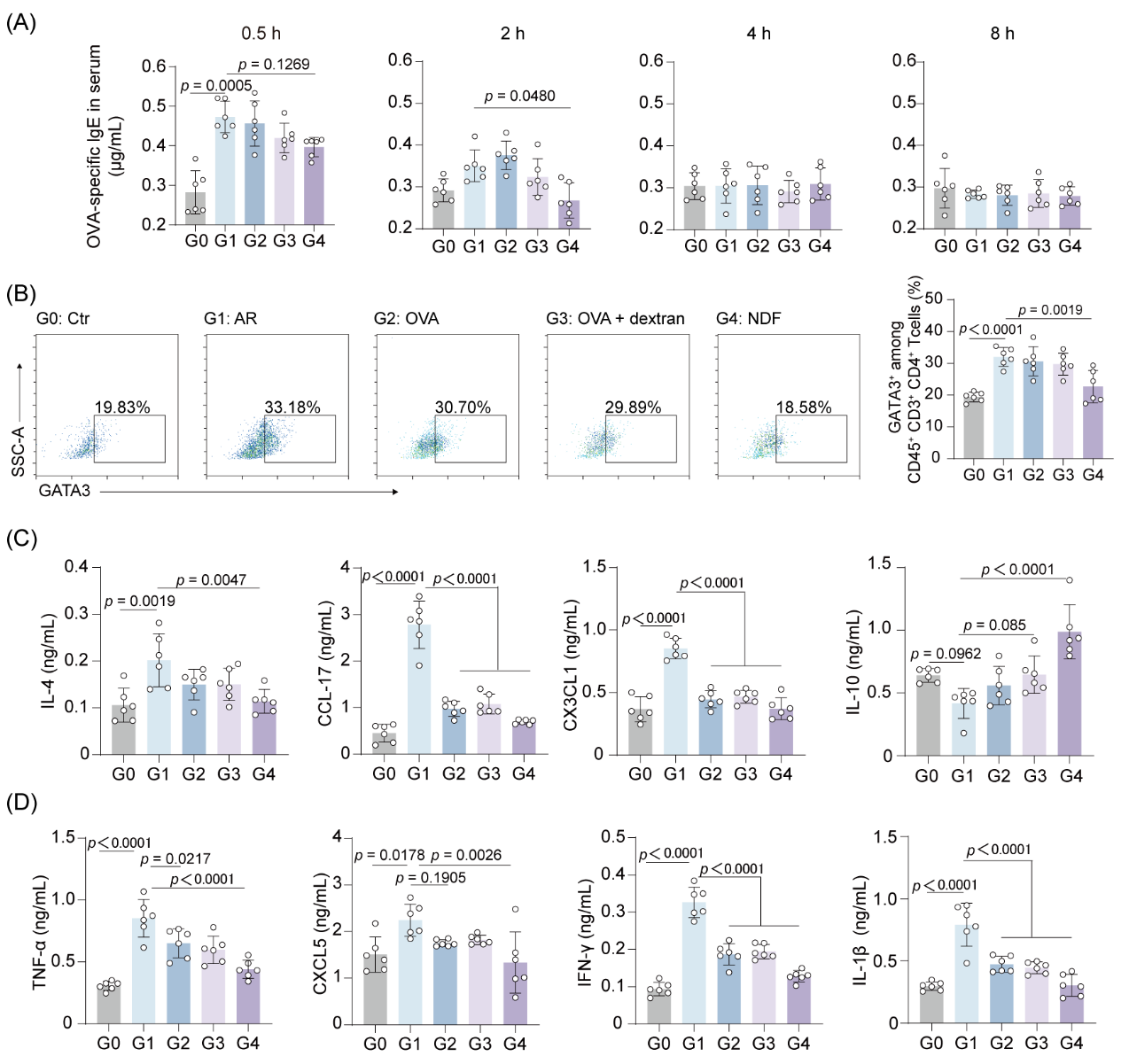


**Figure S4 NDF suppresses local Th2 responses and systemic allergic inflammation. (A) Serum OVA-specific IgE levels at the indicated time points following the final allergen challenge. (B) Flow cytometric analysis of nasal mucosal Th2 cells, presented as the percentage of GATA3⁺ cells among CD4⁺ T cells. (C) Serum levels of the Th2 cytokine IL-4, the regulatory cytokine IL-10, and the chemokines CCL17 and CX3CL1. (D) Serum levels of the inflammatory cytokines TNF-α, IFN-γ, and IL-1β, as well as the chemokine CXCL5.**

**
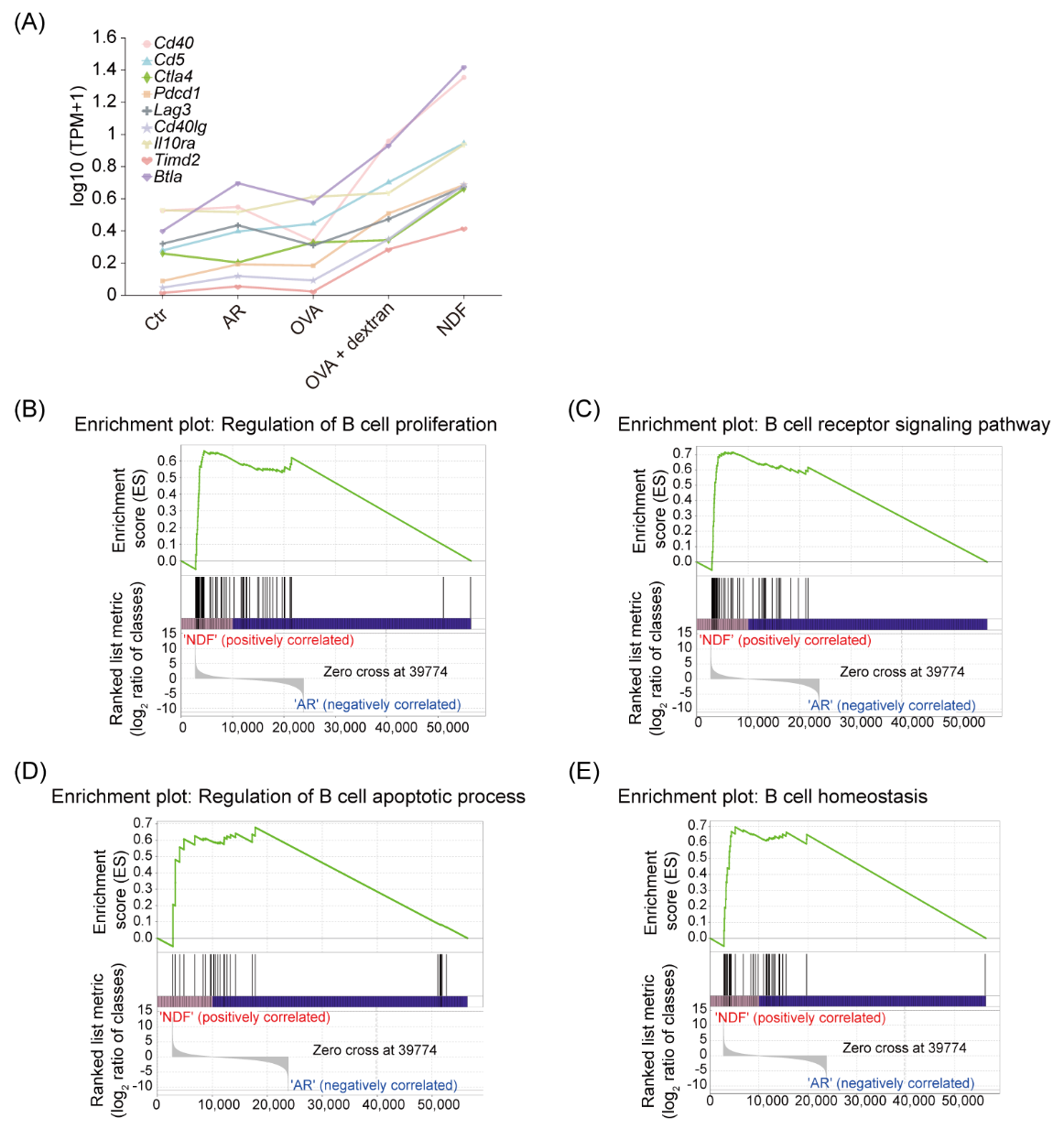
**

**Figure S5 NDF promotes immunoregulatory gene expression and B cell–associated pathways in the intestine.** **(A) Expression of representative immunoregulatory genes (e.g., *Cd40, Cd5, Ctla4, Pdcd1, Lag3*) in intestinal tissues across treatment groups. (B**–**E) GSEA showing enrichment of B cell–related pathways in intestinal tissues from NDF-treated mice compared with AR controls, including regulation of B cell proliferation (B), B cell receptor signaling (C), regulation of B cell apoptotic processes (D), and B cell homeostasis (E).**

**
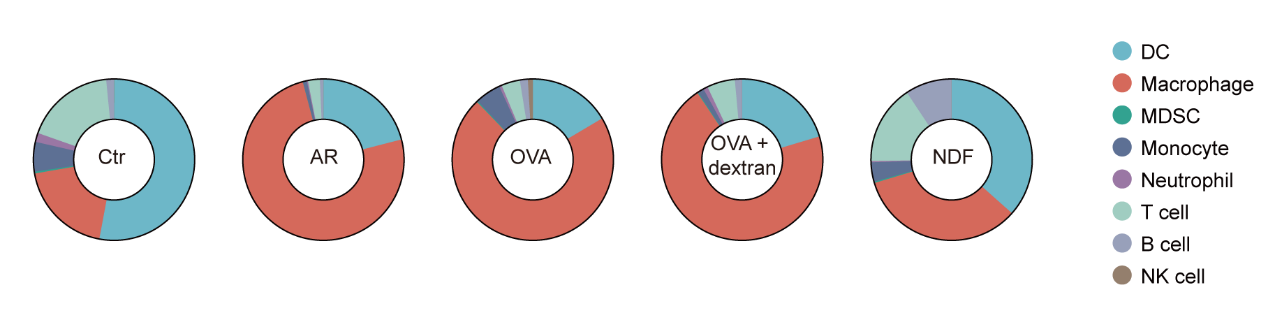
**

**Figure S6 CyTOF profiling of intestinal lymphocyte subsets at endpoint. Mass cytometry (CyTOF) analysis was performed on intestinal immune cells isolated from different treatment groups at the study endpoint.** Pie charts show the relative proportions of major lymphocyte subsets within each group.

**
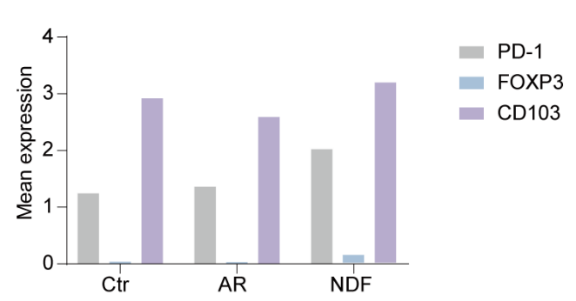
**

**Figure S7 Increased expression of tolerogenic markers in intestinal immune cells following NDF treatment. CyTOF quantification of PD-1, FOXP3, and CD103 expression in intestinal cells from control, AR, and NDF-treated mice.**


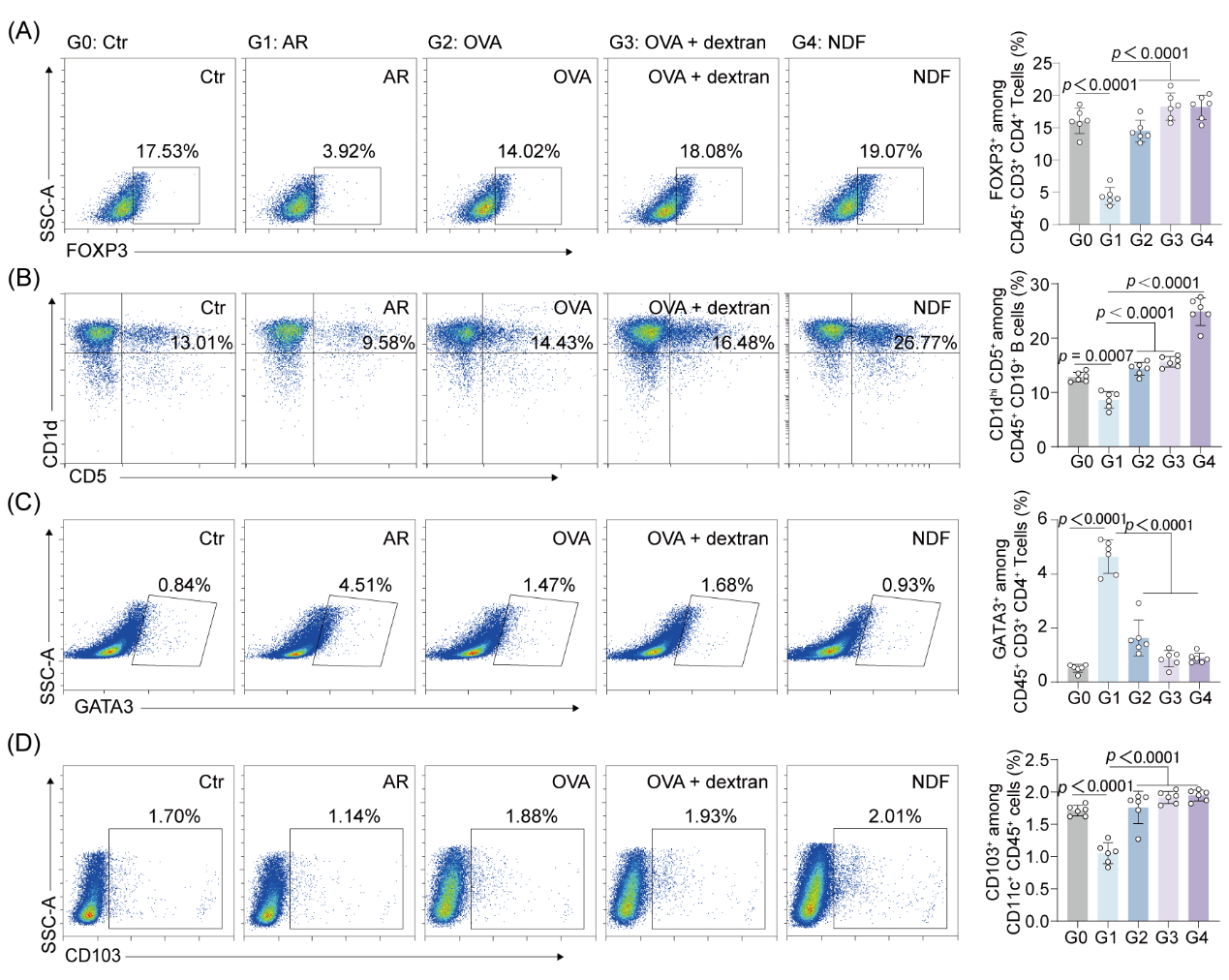


**Figure S8 NDF reprograms the intestinal immune landscape toward a regulatory phenotype. (A–D) Flow cytometric analysis of intestinal immune cell subsets. Representative plots and quantification of** FOXP3^+^**CD4^+^ regulatory T cells (Tregs) (A),** CD1d^hi^CD5⁺ **regulatory B cells (Bregs) (B), GATA3^+^CD4^+^ Th2 cells (C), and CD103^+^ tolerogenic dendritic cells (DCregs) among CD45^+^CD11c^+^ cells (D). Quantitative analyses are shown in the right panels.**


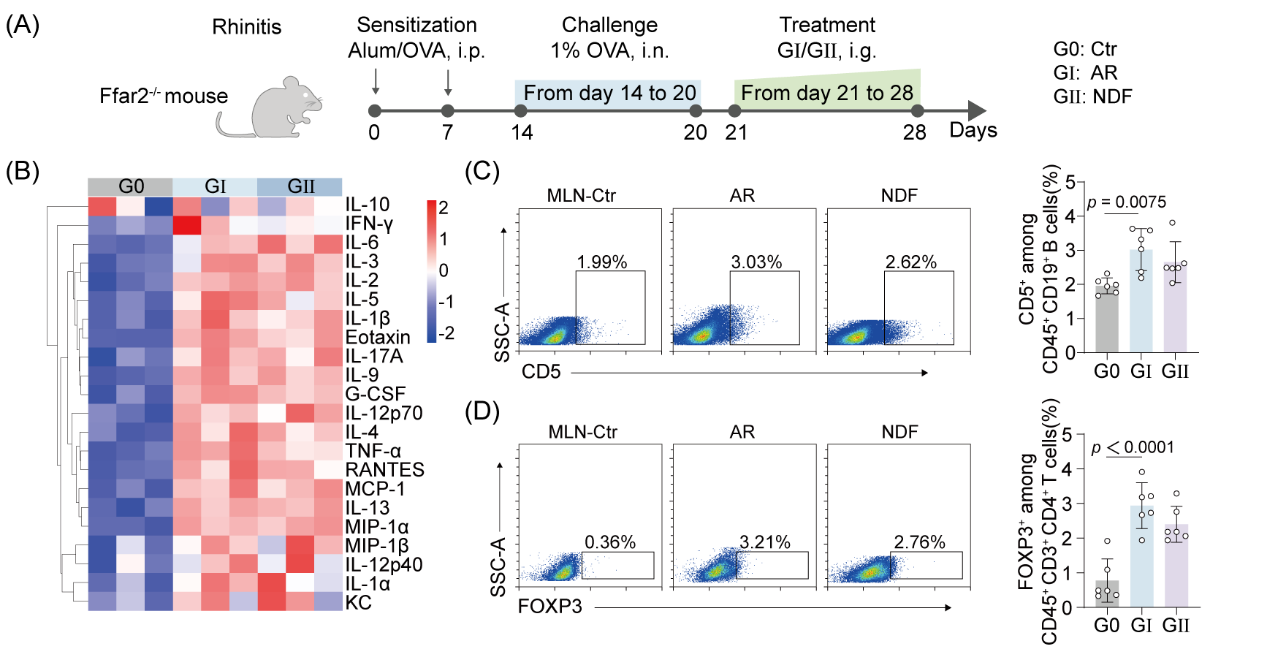


**Figure S9 Comparison of allergic rhinitis induction in *Ffar2^⁻/⁻^* mice*.* (A) Experimental timeline of AR induction and treatment regimen in *Ffar2^⁻/⁻^* mice. (B) Multiplex cytokine analysis of nasal lavage fluid after allergen challenge. (C and D) Flow cytometric quantification of CD5⁺ B cells (C) and FOXP3⁺ T cells (D) in mesenteric lymph nodes.**


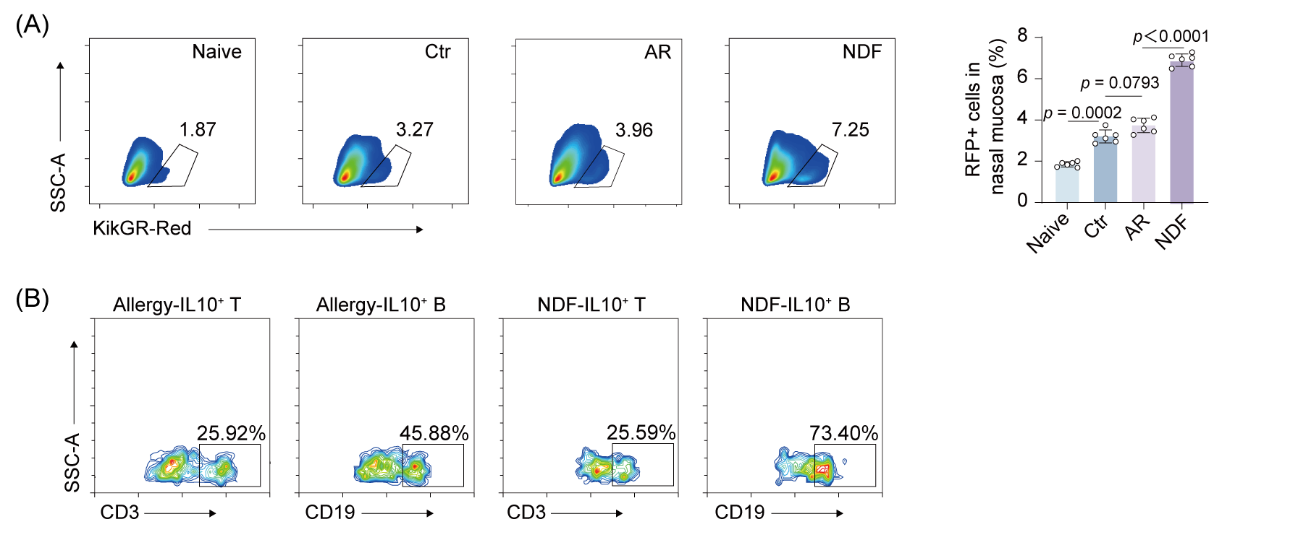


**Figure S10 Gut-derived B cells constitute the major IL-10-producing population in the nasal mucosa. (A) Flow cytometric analysis and quantification of** KikGR-Red^+^ **cells in the nasal mucosa from different treatment groups, showing the migration of photoconverted gut-derived cells**. **(B)** Flow cytometric analysis of CD19^+^ B cells and CD3^+^ T cells among IL-10^+^ cells isolated from the nasal mucosa of AR and NDF-treated mice.


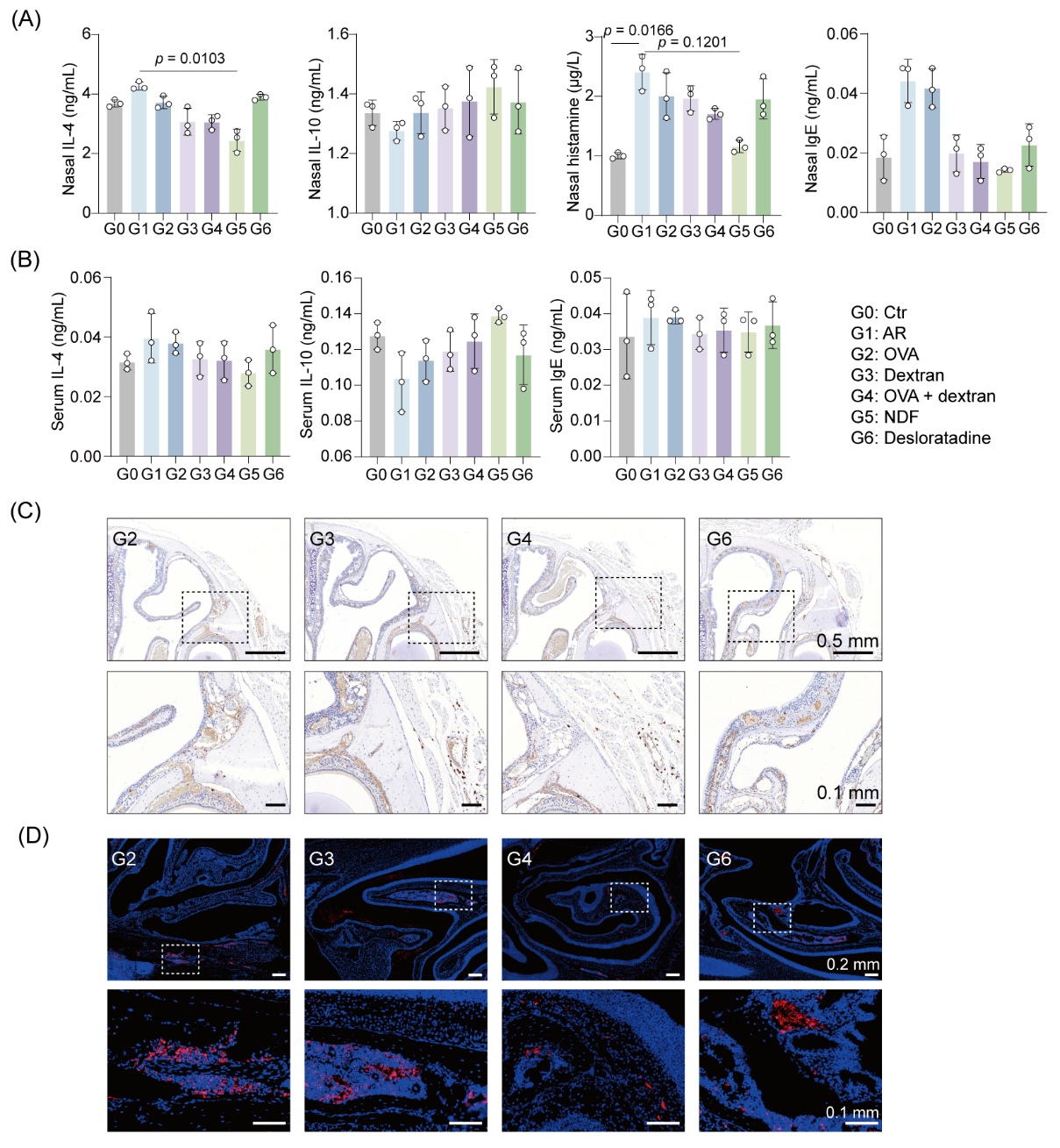


**Figure S11 Cytokine profiles and cellular analysis in nasal tissues post-allergen challenge. (A) Multiplex cytokine profiling of nasal lavage fluid following allergen exposure, measuring IL-4, IL-10, histamine, and IgE levels. (B) Multiplex cytokine profiling of serum post-allergen challenge, including IL-4, IL-10, and IgE levels. (C) Representative immunohistochemical staining of mast cell tryptase in nasal tissues after treatment, accompanied by magnified images (scale bars: top, 0.5 mm; bottom, 0.1 mm). (D) Representative immunofluorescence staining of GATA3 in nasal tissues to evaluate Th2 cells after treatment, with magnified views (scale bars: top, 0.2 mm; bottom, 0.1 mm).**


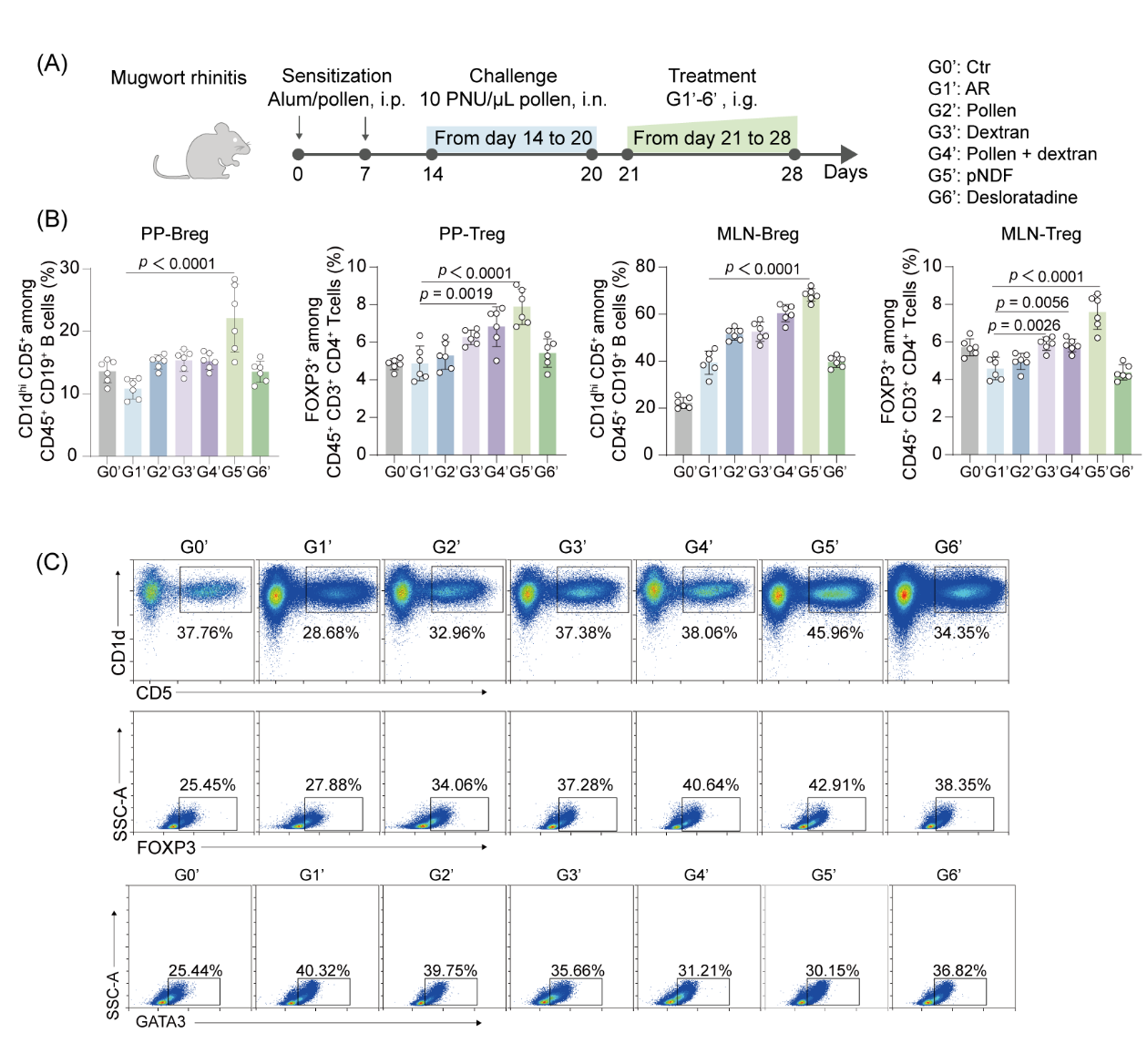


**Figure S12 Immunomodulatory and therapeutic effects in murine models of mugwort-induced rhinitis. (A) Schematic illustration of the mugwort pollen-induced allergic rhinitis model and treatment protocol. BALB/c mice were sensitized by intraperitoneal injection of mugwort pollen extract adsorbed to aluminum hydroxide on days 0 and 7, followed by daily intranasal challenges from days 14 to 20. Therapeutic treatments (PBS, free pollen extract, dextran, pollen–dextran mixture, pNDF, or desloratadine) were administered orally from days 21 to 28 using an escalating pollen dose regimen. (B) Quantification of CD1d^hi^CD5^+^ regulatory B cells (Bregs) and FOXP3^+^CD4^+^ regulatory T cells (Tregs) in mesenteric lymph nodes (MLNs) and Peyer’s patches (PPs). (C) Representative flow cytometry plots of splenic immune cell populations, including CD1d^hi^CD5^+^ Bregs (upper), FOXP3^+^CD4^+^ Tregs (middle), and GATA3^+^CD4^+^ Th2 cells (lower), following mugwort pollen challenge.**


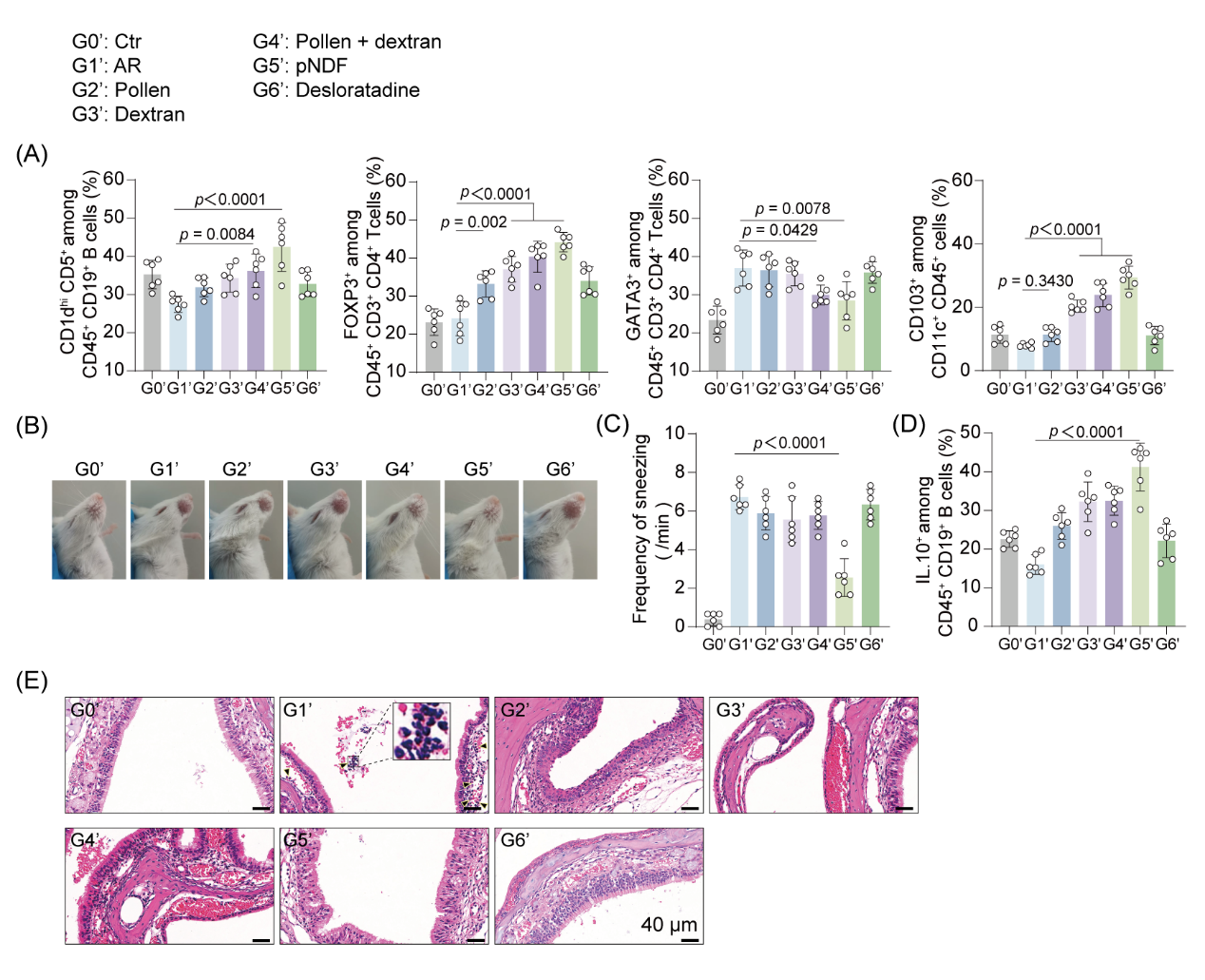


**Figure S13 pNDF promotes regulatory immune responses and alleviates mugwort pollen-induced allergic rhinitis. (A) Quantification of splenic immune cell subsets, including CD1d^hi^CD5^+^ regulatory B cells (Bregs), FOXP3^+^CD4^+^ regulatory T cells (Tregs), GATA3^+^CD4^+^ Th2 cells, and CD103^+^ tolerogenic dendritic cells (DCregs). (B) Representative peri-nasal appearance following pollen challenge. Pollen-challenged mice exhibited alopecia and erythema, whereas pNDF-treated mice maintained a normal appearance. (C) Sneezing frequency recorded within 15 min after the final intranasal pollen challenge. (D) Flow cytometric analysis of IL-10-producing B cells in the nasal mucosa following pollen challenge. (E) Representative histological sections of nasal mucosa from the indicated groups. Black arrowheads indicate inflammatory granulocyte infiltration. Scale bar, 40 μm.**


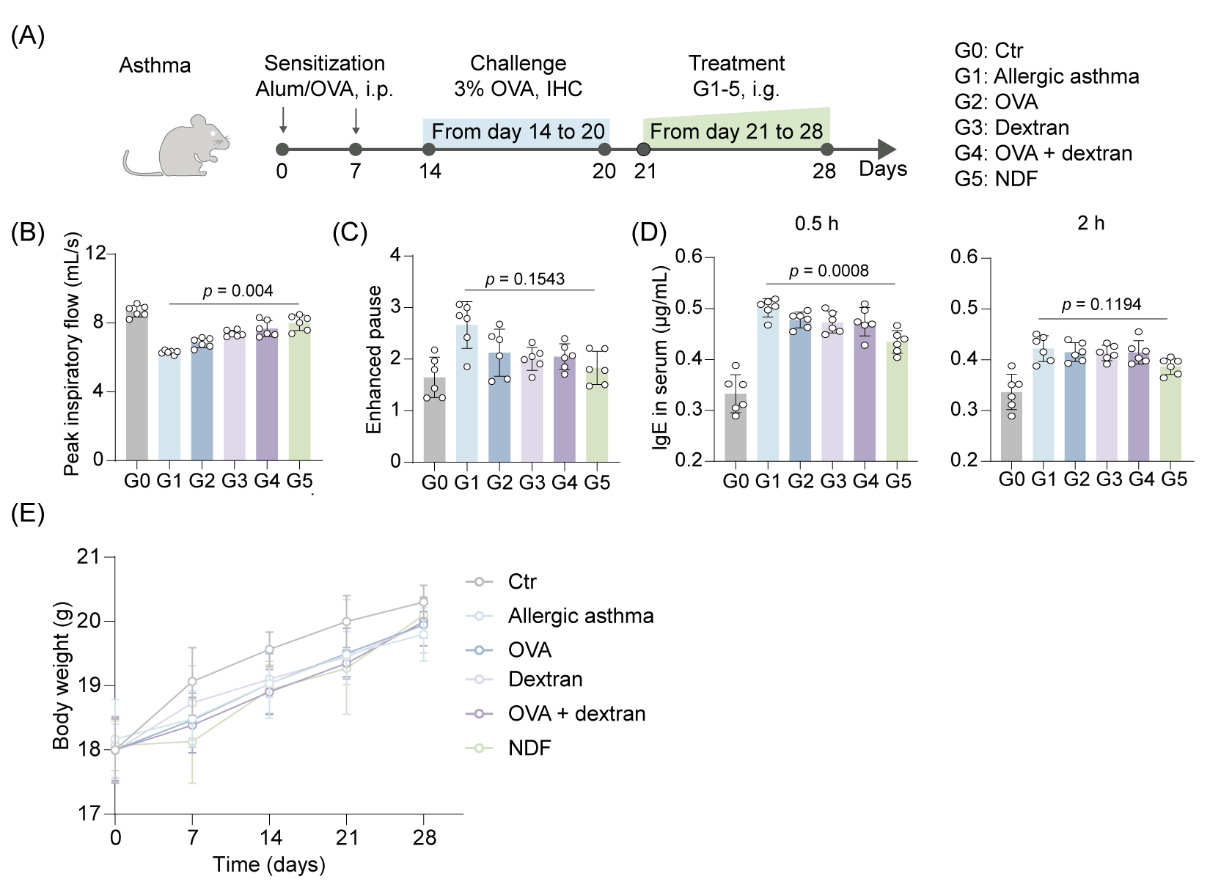


**Figure S14 NDF alleviates allergic asthma and improves pulmonary function. (A)** Schematic illustration of the OVA-induced allergic asthma model and treatment protocol. Mice were sensitized with OVA and alum on days 0 and 7, followed by daily aerosolized OVA challenges from days 14 to 20. Oral treatments (PBS, OVA, dextran, OVA plus dextran, or NDF) were administered from days 21 to 28 using an escalating OVA dose regimen and a fixed dextran dose. IHC, inhalation challenge. (B–C) Pulmonary function measurements, including peak inspiratory flow (PIF, B) and enhanced pause (Penh, C), in the indicated treatment groups. (D) Serum OVA-specific IgE levels measured 0.5 h and 2 h after the final allergen challenge. (E) Body weight changes during treatment in mice with allergic asthma.
